# Supplementary material for: Student intentions to continue with distance learning post-COVID: An empirical analysis
Source: PLoS One. 2024 Jan 17;19(1):e0293065. doi: 10.1371/journal.pone.0293065 (PMC10793936; doi:10.1371/journal.pone.0293065)
Supplement: S1 Appendix — (DOCX) [file pone.0293065.s001.docx]

S1 Appendix – Scale Items and Sources

| Scale | Items | Source(s) |
| --- | --- | --- |
| D/L continuance intentions | When no longer required to do so, I would take distance learning classes in the future. | [46] |
|  | Taking distance learning classes is something I would do when no longer required to do so. |  |
|  | I could see myself taking distance learning classes when no longer required to do so. |  |
| Satisfaction | I would recommend online classes to other students. | [65] |
|  | I am satisfied with the quality of the learning experience via the online class. |  |
|  | I enjoyed online classes |  |
| Faculty support | My professors really care about me. | [8] |
|  | My professors are willing to help me when I need it. |  |
|  | I can rely on my professors. |  |
|  | When I have questions my professors are available. |  |
| Technical support | Adequate technical support is available from my school. | [66] |
|  | The technical support provided by my school is good. |  |
|  | When I need technical support, I am able to get it. |  |
| Institutional support | Help is available from my school when I have a problem. | [67] |
|  | My school really cares about my well-being. |  |
|  | My school is concerned about me as a person. |  |
| Technical compatibility | I believe that it is easy to get online learning technologies to do what I want them to do. | [65] |
|  | Overall, I believe that distance learning technologies are easy for me to use. |  |
|  | I had no trouble with the technology. |  |
| Environmental compatibility | My living conditions during the term were suitable for online classes. | [16] |
|  | I could organize my room/work area to facilitate online learning. |  |
|  | I had no trouble learning in my home environment. |  |
| Learning compatibility | Taking distance learning classes fits my preferred way of learning. | [46] |
|  | Distance learning enables me to learn in the way I prefer. |  |
|  | Distance learning fits well with the way I like to learn. |  |
|  | Distance learning fits my preferred method for learning. |  |
