# Supplementary material for: Student intentions to continue with distance learning post-COVID: An empirical analysis
Source: PLoS One. 2024 Jan 17;19(1):e0293065. doi: 10.1371/journal.pone.0293065 (PMC10793936; doi:10.1371/journal.pone.0293065)
Supplement: Attachment — Submitted filename: 2 Response to Reviewers.docx [file pone.0293065.s003.docx]

PLOS ONE Decision: Revision required [PONE-D-23-06971] - [EMID:febd35bad5c1e838

| Comment | Response |
| --- | --- |
| AE |  |
| Here is a summary of their [the reviewers’] suggestions:  1. Improve the abstract, results section, and discussion of findings.  2. Strengthen the introduction, methodology, and discussion sections.  3. Enhance the literature review and theoretical support.  4. Include more studies related to student intentions to continue with distance learning.  5. Assess and address common method bias.  6. Address issues with HTMT values, research model, and measurement items.  7. Revise the implications section to provide specific and actionable suggestions.  8. Elaborate on the significance of the study in the conclusion section.  9. Address limitations and future work.  10. Proofread the manuscript for typos and mistakes. | Thank you for summarizing the reviewers’ comments. We have attempted to address each of them, as detailed below. |
| RV 1 |  |
| Further the introduction is a bit weak. Why is the research needed now? Look at how to create a compelling problematization and a hook for readers to be engaged in your study. Problematization is extremely important to showcase why your findings are going to be useful for explaining the phenomenon in the future. Explicitly introduce research questions / research objectives towards the end of the introduction, as bullet points, before sharing how the remaining article is structured | Thank you - we have added a paragraph to address this and indicated some of the problems facing university administrations and students. We have also strengthened the need for studies such as this to be done now while changes to higher education are high in the minds of administrators.  Done - we have included just one broad RQ - this is further developed into hypotheses in the next section. |
| The research methodology needs strengthening. Can you introduce subsections for data collection and data analysis methodologies separately? What could have been trade-offs or biases in your study? Particularly, define the population of the study and sampling technique adopted. Also please justify why your chosen sampling technique and sample size are appropriate. | Thank you - we have introduced the requested subsections, defined the target population and sampling technique and justified the sample size. |
| The discussion part can be broadened a bit more with more relevant research studies, as we know there are huge volume of research in this area. | Thank you, we have incorporated more studies to the paper as a whole. |
| I found the implications section to be somewhat weak, as there is a lack of clear connection between the results of the quantitative data analysis and practical implications. The authors should consider revising the implications section to provide more specific and actionable suggestions for practitioners, developers, and designers of metaverse-based learning platforms in higher education. | Thank you, we have improved our discussion of the implications of the study in the abstract and in the discussion section. |
| The literature review, the theoretical support, and the discussion sections can benefit from well-established related research, including: -Developing a holistic success model for sustainable e-learning: A structural equation modeling approach. https://doi.org/10.3390/su13169453 - Novel extension of the UTAUT model to understand continued usage intention of learning management systems: The role of learning tradition. https://doi.org/10.1007/s10639-021-10758-y - Exploring Student Readiness to MOOCs in Jordan: A Structural Equation Modelling Approach. https://doi.org/10.28945/4542  - Towards a Sustainable Adoption of E-Learning Systems: The Role of Self-Directed Learning. https://doi.org/10.28945/4980 | Thank you for drawing our attention to your papers - we have incorporated several into our paper… in two areas. For those papers that are relevant to the study and were published prior to our development of the research model and the resultant survey we have included them in the literature review to support our satisfaction issues and for those published after model development we have incorporated some of the issues in our discussion section. |
| RV 2 |  |
| The manuscript needs a proof reading. The author(s) should check English since there are (much too) many instances of typos and mistakes throughout the manuscript. | Thank you - this has been done. |
| The authors claims that “All the HTMT values were lower than the conservative threshold of 0.85.” However, the HTMT value between 2 and 8 in Table 3 is 0.867. | Thank you for picking this up - we have modified the text to indicate the one minor transgression. However, as stated in the sentence that followed, based on a bootstrapping procedure, this one minor exception and others did not have a HTMT value statistically different from 1. In addition, we added a brief note that 0.85 is a strict heuristic; there is another well-established heuristic that indicates 0.90 is an acceptable cut-off for establishing discriminant validity. |
| Figure 2 provides a research model showing the relationships between study variables. Please use a dashed line to indicate nonsignificant paths. The items measuring each dimension in the model were provided. Several items are not relevant to the study context (distance learning). For example, “My professors really care about me; I can rely on my professors; My school really cares about my well-being; My school is concerned about me as a person.” Each item should be related to distance learning. | We have revised Figure 2 as requested.  Regarding the relevance of the scales, we did make the support scale items more general (not specific to distance learning). We did this because we think that students would have trouble separating out distance learning specific supportiveness from overall supportiveness. We explain our reasoning on p. 16 in the following text.  *“The wordings for the support scales are more general; we left these general because it is difficult for students to separate general support from support that is specific for distance learning. For example, when evaluating whether their university is supportive, it is difficult for students to parse out distance learning and other aspects of their university experience. We worded the support items to be more general.”* |
| Further, as the hypotheses were related to the study context “e.g., the perceived institutional support for distance learning will positively influence students’ satisfaction with distance learning” each measurement item should be tailored to the study context. | We apologize for this inconsistency. According to organizational support theory, individuals form global beliefs about the extent to which an organization cares about their well-being. It is difficult for organization members to parse out beliefs about support to specific, discrete aspects of their experience as organizational members. Therefore, the support scales are worded to reflect global beliefs rather than distance learning specific beliefs. We have added a paragraph explaining this logic and have revised H2a, b, and c to reflect our thinking. |
| Descriptive Statistics (Mean, S.D., Skewness, Kurtosis) for each subdimension should be reported. | We have added this information. Please see Table 2. |
| Since this is a cross-sectional study, common method bias may arise from certain tendencies that respondents apply. Therefore, common method bias should be an important concern and assessed. | We took several steps related to common method bias. First, apriori we varied scale endpoints, which can help reduce common method variance (CMV). We also ran a full collinearity assessment, which indicated that no problematic CMV was present. In addition, we conducted a marker variable analysis, which also indicated that CMV was not a serious problem. Please see the subsection “Common method bias” on p. 17 for details. |
| Discussion & Conclusion should be enlarged, including a better interpretation of results and a better justification of the conclusions. Refer to the following relevant articles in the revised manuscript: “Generation Z use of artificial intelligence products and its impact on environmental sustainability: A cross-cultural comparison”, “Examining the impact of psychological, social, and quality factors on the continuous intention to use virtual meeting platforms during and beyond COVID-19 pandemic: A hybrid SEM-ANN approach”, “The impact of knowledge management practices on the acceptance of Massive Open Online Courses (MOOCs) by engineering students: A cross-cultural comparison”, “The role of self-efficacy in predicting use of distance education tools and learning management systems”, and “The role of self-directed learning, metacognition, and 21st century skills predicting the readiness for online learning.” | Thank you for this useful list of additional references. Several of them have provided a useful addition to our literature review and we have incorporated them. |
| RV 3 |  |
| The abstract does not discuss on data collection and data analysis tools. The key findings are not well written. | Thank you - we have tightened the abstract to make it clear the data relates to South Africa and that PLS was applied to a developed model. We have also made a more specific summary of the key findings. |
| The key findings are not well written. Thus, improve the main issue to be addressed in the study and finding part of the abstract. | Thank you - the key findings have been expanded upon and a summary included in the abstract. |
| The introduction section needs to be improved to include the main research problems and research questions. | Thank you - this has been done. See the comments on the Introduction in the section dealing with Reviewer 1 above. |
| Included studies related to Student intentions to continue with distance learning such as; Alarabiat, A., Hujran, O., Soares, D., & Tarhini, A. (2023). Examining students' continuous use of online learning in the post-COVID-19 era: an application of the process virtualization theory. Information Technology & People, 36(1), 21-47.  Anthony Jnr, B., & Noel, S. (2021). Examining the adoption of emergency remote teaching and virtual learning during and after COVID-19 pandemic. International Journal of Educational Management, 35(6), 1136-1150.  Marandu, E. E., Mathew, I. R., Svotwa, T. D., Machera, R. P., & Jaiyeoba, O. (2023). Predicting students' intention to continue online learning post-COVID-19 pandemic: extension of the unified theory of acceptance and usage technology. Journal of Applied Research in Higher Education, 15(3), 681-697.  Jnr, B. A., Kamaludin, A., Romli, A., Raffei, A. F. M., Phon, D. N. A. E., Abdullah, A., ... & Baba, S. (2020). Predictors of blended learning deployment in institutions of higher learning: theory of planned behavior perspective. The International Journal of Information and Learning Technology, 37(4), 179-196. | Thank you for this useful list of additional references too. Again, several of them have provided a useful addition to our literature review and we have incorporated them. |
| What sampling methods was employed in the study. Also, provide justification on the employed methodology (statistical tool if any e.g) utilized in the study. | We employed a convenience sample drawn from two major SA institutions. We have amended the methodology section to reflect this. We have also added a sentence to the Limitations section.  Justification for using has been explained in the 2nd paragraph of the **Analysis and Results** section |
| How was the reliability and validity assessed for this study. This should be well discussed based on existing scales or benchmark from the literature. | We analyzed the measurement model corresponding to our research model to assess reliability and validity. We followed standard, well-established procedures and benchmarks using statistics including Chronbach’s alpha, composite reliability, average variance explained, among others. Please see the subsection, Measurement model, which includes relevant reliability and validity information in Tables 3 and 4. |
| Improve the results section and related back to the literature. What are the main discoveries from the study, can you summarize as a model (figure) or table | Please see Figure 2 and Table 5 for summaries of the main findings. The hypotheses numbers in the figure and table refer back to the theoretical development section, which references relevant prior literature. |
| I miss a comparison of your results as compared to other studies in the literature on Student intentions to continue with distance learning. | Thank you - several of the papers recommended by you and other reviewers have provided further information on this (including one from a neighboring country) and we have incorporated these into the paper. |
| The discussion section needs to discuss findings from the literature and also findings from the current study. Also, include the research implications of the study as a different section. This can be implication for theory as well as the implication for practice/policy | Thank you - the discussion section has been expanded, as well as the implications of the study for University practice, for students and also a contribution to knowledge. |
| The conclusion section should elaborate more on the significance of the study. Lastly improve the limitations and future works | Thank you - the significance of the study has been elaborated upon, with limitations now also included in the study. |
| In particular, what is the main contribution from this study it’s hard to see OVERALL, write manuscript as introduction, literature review, methodology, findings, discussion and implications, conclusion. | Thanks. The main contribution of the study has been emphasised in the introduction as well as in the conclusions. |
